# “Why don’t we just add a camera?”: a psycho-genetic perspective on precision livestock farming in pigs

**DOI:** 10.1186/s40813-024-00402-9

**Published:** 2024-11-22

**Authors:** Carmen Winters, Wim Gorssen

**Affiliations:** 1 Animal Physiology, Institute of Agricultural Sciences, ETH Zurich, Universitätstrasse 2, Zürich, 8092 Switzerland; 2 Center for Animal Breeding and Genetics, Department of Biosystems, KU Leuven, Kasteelpark Arenberg 30, Box 2472, Leuven, 3001 Belgium; 3 Animal Genomics, Institute of Agricultural Sciences, ETH Zurich, Universitätstrasse 2, Zurich, 8092 Switzerland

*“Why don’t we just add a camera?”* This seemingly simple question sparked our unconventional collaboration and eventually led us to co-edit a special collection on Precision Livestock Farming (PLF) in pigs. What initially seemed like an unlikely partnership between a biological psychologist (Carmen) and a geneticist (Wim) became a perfect fit, as our differing perspectives converged on the same experiment. While genetics determines the biological predispositions of pigs, behavioral psychology provides insights into how these predispositions interact with environmental stimuli to shape behavior and welfare. As such, we firmly believe that a multidisciplinary approach is essential to advancing the dynamic and evolving field of PLF. The future of pig farming lies in the integration of insights from various disciplines.

PLF emerged from the intersection of agricultural sciences, computer sciences and biology, and its development over the past decades has been remarkable [1, 2]. A core goal of PLF is to improve animal welfare, particularly by early detection and even prevention of illness and injury. However, we believe the primary challenge facing pig production today extends beyond production efficiency and animal welfare; it lies in addressing public perceptions of pig farming.

Public consciousness has sparked debates over existing practices and led to practical changes in tail docking, castration, and crating sows in Europe. As society’s values evolve, so must farming practices and technological solutions. Pigs are no longer viewed as mere “walking bacon” but are increasingly recognized as sentient, intelligent creatures capable of complex social interactions. Although PLF provides valuable tools to enhance welfare, we must remain grounded in reality. It cannot guarantee a perfect life for every animal, nor does it change the fundamental purpose of pig farming: meat production. Expecting every pig to live a stress-free, content life is unrealistic and arguably even adverse to general welfare [3]. Even with the best interventions, animals will face challenges, and ultimately, their fate is to be slaughtered for food. Holding PLF to a standard where it guarantees a completely stress-free existence for livestock is unrealistic, especially given that humans have yet to achieve such conditions for themselves. We need to set realistic goals to avoid disillusionment. By combining accurate phenotyping methods with ethical breeding practices, we can improve pig welfare meaningfully without overreaching.

Technological advancements enable continuous data collection from livestock using a variety of sensors. These tools provide unprecedented precision, allowing farmers and researchers to monitor individual animals rather than whole groups. As a result, we can capture changes in behavior within specific environments and across generations, opening new doors for research and practical applications. These data can also be easily stored, shared, and reused, which expands their value far beyond the initial research goals. Despite these advancements, the real-world impact of PLF on pig welfare and health remains limited. Pig farmers are still facing challenges, infectious diseases and harmful behaviors such as tail biting and aggression. With this collection, we aim to raise critical questions and offer a platform for researchers to explore how PLF can help address these issues.

PLF offers new ways to adapt the animals themselves via selective breeding. By integrating genomic selection with data collected from PLF technologies, we can breed pigs that are more resilient to environmental challenges and less prone to harmful behaviors [4]. Genomic selection revolutionized breeding practices in the 2000s [5], but phenotyping has become a bottleneck in the process [6]. Fortunately, advancements in PLF, such as computer vision systems, are helping to close this gap. Our own research [7] demonstrated how PLF could estimate pigs’ activity levels during weighing, revealing moderate heritability estimates for behavioral traits. In general, behavioral traits are proving to be more heritable than previously thought, which opens the door to breeding pigs for specific behavioral characteristics. However, this must be approached carefully to avoid unintended consequences, such as breeding pigs that are overly docile or apathetic.

For a behavioral psychologist, the increasing potential of PLF technologies to monitor subtle behavioral markers is fascinating. In pigs, (chronic) stress triggers a cascade of hormonal and neural responses, which can escalate into harmful behaviors, such as tail biting [3]. Detection of behavioral changes in movement, vocalization or social interactions in response to stressors or environmental stimuli could enable early interventions before any harm is done. By using PLF to monitor specific behaviors, such as cooperation, fear or aggression, we could breed pigs that are not only physically robust but also psychologically resilient. This form of selection, guided by both genetic and psychological knowledge, could lead to healthier, more socially stable pig populations. By focusing on pigs that show resilience to stress, we can breed animals that are not only healthier but also more adaptable to changing environments. Insights into how these traits are processed in the brain could provide a deeper understanding of what makes some pigs more resilient than others, allowing for more targeted breeding and management strategies.

Ultimately, we need more fundamental research to understand what “improved welfare” means for pigs, and not how it is merely perceived by humans. Our knowledge of pig behavior and its underlying mechanisms is still developing with many scientific gaps to be filled in. Similarly, breeding for positive social behaviors could enhance welfare, but we must be mindful not to anthropomorphize pigs or expect them to conform to human (psychological) constructs such as happiness. As a result, we lack research-based management techniques that work for both animals and farmers.

We invite researchers from all disciplines to contribute to this collection on PLF in pigs. We are convinced that this ensemble approach is needed and the best way to move forward and come up with new solutions for the current challenges in pig production. With joint forces, let us make pig production great again!
